# Fracture of the Clavicle

**Published:** 1855-12

**Authors:** F. H. Hamilton


### ART. V.—*Fracture of the Clavicle.* By Prof. F. H. Hamilton.

Doctor Hunt:

Dear Sir,—Will you do me the favor to call the attention of your readers, and especially of the Editors of other medical journals, to an error in the "Transactions of the American Medical Association" just published, for which, however, I wish to say, the publishing committee are not responsible.

On page 436, 19th line from the top, substitute for the word able, *unable;* so that it shall read "it is unable to prevent a riding of the fragments."

If you cannot use your space more profitably you may add, also, the following opinions as to the prognosis in fractures of the collar-bone.

Very truly yours,

Frank H. Hamilton.

"As to the Reduction of this *Fracture*, it must be own'd the same is often easier replaced, than retained in its place after it is reduced: For its Office being principally to keep the Head of the *Scapula*, or Shoulder, to which at one end it is articulate, from approaching too near, or falling in upon the *Sternum*, or Breast-Bone, it happens, that on every Motion of the Arm, unless great Care be taken, the *Clavicle* therewith rising and sinking, the fractured Parts are apt to be distorted thereby. Besides, even in the common Respiration, the *Costæ* and *Sternon* aforesaid, where the other end of this Bone is adnected, together with the Motion of the *Diaphragm* rising and falling, especially if the same be extraordinary, as in Coughing and Sneezing, are able to undo your Work: Not to mention the Situation thereof, less capable of being so well secured by *Bandage* as many others. All which duly considered, 't is no wonder that upon many of these Accidents altho' great Care has been taken, these Bones are sometimes found to ride and a protuberance is left behind, to the great regret particularly of the Female Sex, whose Necks lie more exposed, and where no small Grace or Comliness is usually *Placed*." * * * * * *.— *The Art of Surgery, by Daniel Turner. Vol.* 2, *p.* 256. *London Ed.* 1742.

"Restituitur facile tractis humeris a ministro posterius, dum simul suo genu locato ad Spinam dorsi, dorsum sustentet minister, nam tunc Chirurgus folis digitis claviculam fractam reponere potest. *Difficilius autem in reposita sede retinetur*, sed loca cava supra et infra claviculam spleniis implenda;"—*Johannis de Gorter*: *Chirurgia Repurgata*, *p.* 79. *Lugduni Batavorum.* 1742.

"The Reduction of a broken Clavicle is not very hard to be effected, especially when the Fracture is transverse; nor is it usual for the *Humerus*, with the Fragment of the Clavicle, to be so far distorted as not to be easily replaced with the Fingers: *but the Difficulty is much greater to keep the Bone in its Place, when the Fracture is once reduced, especially if the Bone was broken obliquely*."—*Heister's Surgery*, *vol.* 1, *p.* 134. *London Ed.* 1768.

Amesbury, after having exposed the inefficacy of all previous modes of dressing, and especially of the figure of 8 bandage, Desault's, Boyer's, and an apparatus recommended by Sir Astley Cooper, proceeds to describe his own apparatus and to affirm its excellence. It is, however, not much unlike a multitude of others, and is liable, I have no doubt, to the same objections. But the author thus writes:

"The clavicle-bandage once properly applied, enables the surgeon to resist the action of all those powers which tend to produce displacement, and puts the fractured bone entirely under his control, without being productive of any of those evils which, I have endeavored to show, arise from the usual modes of treatment. I am not prepared to say that every fracture of the clavicle, treated in the manner which I have thought it expedient to advise, admits of being united without deformity; but, I am fully convinced that, if such cases should occur, they will be found very rare. I have had this bandage in use now about eight years, and, in the course of this time, I have used it in a large number of cases, many of which have occurred in the St. Thomas Hospital. The result of these cases has been very satisfactory to myself, and to those surgeons by whom the treatment was witnessed."—*Treatment of Fractures, by Joseph Amesbury, vol.* 2, *p.* 527. *London Ed.* 1831.

"The direction the fracture takes is generally oblique, and in one place only, more particularly when it is caused by the indirect force: and this obliquity is one great reason why it is so difficult to treat this kind of injury without some slight deformity *(and often a very great one)* existing afterwards." * * * "One satisfactory result in the treatment of fracture of the clavicle is, that although it is very difficult to prevent deformity afterwards, the motion and free use of the limb do not become much impaired; *for the bone is often shortened an inch or more*, and still the limb possesses free motion and strength. This shortening causes the neck of the scapula to fall forwards, and makes the base of it project backwards, giving the person the appearance of having the chest contracted in front; by which the extent of range of action in the upper extremity will be diminished, although sufficient motion remains for the ordinary uses of the upper extremity. Where the fracture occurs in females, in whose dress the clavicle is exposed to view, it becomes an additional object to get the bone to unite as evenly as possible, to guard against the formation of an unsightly lump that will remain for ever afterwards. The more the deformity is prevented in these cases, the more credit the surgeon will get for the cure."—*Practical Treatise on Fractures, by Edward F. Lonsdale, p.* 207 *and* 209. *London Ed.* 1838.

M. Mayor, of Lausaune, thinks that up to this day no successful mode of treatment had been devised. "Here every thing appears as yet so little determined that each day sees some new propositions and different proceedures," &c. He believes, however, that in his simple handkerchief bandage, with straps across *each* shoulder, the indications are most fully accomplished and the most successful results are obtained. If, however, it were to be treated *without* apparatus, the horizontal position, lying upon the back, would in the end make the most perfect unions.—*Nouveau Systeme de Deligation Chirurgicale, par Mathias Mayor de Lausaune, p.* 384, *etc., (also Atlas, plate* 3, *fig.* 23.*) Paris Ed.* 1838.

Says M. Malgaigne, "The prognosis, considering the trivial character of this fracture, is sufficiently difficult. For, little as may be the displacement, the surgeon ought not to promise a reunion without deformity; and certain successful results, proclaimed from time to time, betray, on the part of those who relate them, the most extravagant exaggerations."—*Traité des Fractures et des Luxations, par J. F. Malgaigne. Tom. premiere, p.* 473. *Paris Ed.* 1847.

M. Nelaton having spoken of the various plans which have been suggested to retain this bone in place, and of their inefficiency, comes at least to speak of the handkerchief bandage of M. Mayor, and remarks:

"This apparel is very simple; but neither will it remedy the overlapping." * * * * "Of all the apparels which we have passed in review, there is, then, not one which fills completely the three indications usually present in the fracture of a clavicle. None of them oppose the displacement; they have no effect, with whatever care they may be applied, but to maintain immobility in the limb. We think, then, that it is useless to fatigue the patient with an apparatus annoying, and, perhaps, even painful; a simple sling, secured upon the sound shoulder will be sufficiently severe. Nevertheless as this does not assure so complete immobility as the bandage of M. Mayor, it is to this that we think the preference ought to be given in all cases of fractures of the clavicle, whether accompanied with displacement or not, whether they occupy the middle or the external part of the clavicle. If the fracture presents no displacement we shall obtain a cure which will leave nothing to be desired. If there is a tendency to displacement, the consolidation will be effected with a deformity more or less marked; *but since this deformity is inevitable, at least with adults, whatever may be the apparel which we employ*, it is evident that the apparatus which causes the least constraint ought to have the preference. We may remark farther, that this union with deformity in nowise impairs the free exercise of all the movements of the member."—*Élémens de Pathologie Chirurgicale, par A. Nelaton, Tom. premiere, p.* 720. *Paris Ed.* 1844.

